# Predictors of oral rotavirus vaccine immunogenicity in rural Zimbabwean infants

**DOI:** 10.1016/j.vaccine.2020.01.097

**Published:** 2020-03-17

**Authors:** James A. Church, Bernard Chasekwa, Sandra Rukobo, Margaret Govha, Benjamin Lee, Marya P. Carmolli, Robert Ntozini, Kuda Mutasa, Monica M. McNeal, Florence D. Majo, Naume V. Tavengwa, Beth D. Kirkpatrick, Lawrence H. Moulton, Jean H. Humphrey, Andrew J. Prendergast

**Affiliations:** a Zvitambo Institute for Maternal and Child Health Research, Harare, Zimbabwe; b Centre for Genomics & Child Health, Blizard Institute, Queen Mary University of London, UK; c Vaccine Testing Center, Department of Pediatrics, Larner College of Medicine, University of Vermont, Burlington, VT, USA; d Vaccine Testing Center, Department of Microbiology & Molecular Genetics, Larner College of Medicine, University of Vermont, Burlington, VT, USA; e Department of Pediatrics, University of Cincinnati College of Medicine, Division of Infectious Diseases, Cincinnati Children’s Hospital Medical Center, Cincinnati, OH, USA; f Department of International Health, Johns Hopkins Bloomberg School of Public Health, Baltimore, MD, USA

**Keywords:** Infants, Africa, Rotavirus, Oral vaccine, Immunogenicity

## Abstract

**Background:**

Oral rotavirus vaccines (RVV) have poor immunogenicity in low-income countries, for reasons that remain unclear. This study identified the determinants of RVV immunogenicity among infants in rural Zimbabwe.

**Methods:**

Anti-rotavirus IgA titres were measured among a sub-group of infants enrolled in the Sanitation Hygiene Infant Nutrition Efficacy (SHINE) trial (NCT01824940). SHINE was a cluster-randomized trial of improved infant and young child feeding, and improved water, sanitation and hygiene (WASH) in two rural Zimbabwean districts. Infants received RVV as part of the national immunisation programme. Among HIV-unexposed infants in the non-WASH trial arms, we evaluated associations between potential risk factors (vaccine schedule and dose, maternal and infant nutritional status, infant diarrhoea, and household environment) and RVV immunogenicity (seroconversion, seropositivity and geometric mean titres) using multivariable regression.

**Results:**

Among 219 infants with seroconversion data, 43 (20%) successfully seroconverted and 176 (80%) failed to seroconvert to RVV. Seroconversion was positively associated with a higher length-for-age Z-score (LAZ) around the time of vaccination (adjusted RR 1.27 (95% CI 1.04, 1.55), P = 0.021), and negatively associated with concurrent OPV and RVV administration (adjusted RR 0.36 (0.19, 0.71), P = 0.003). Among 472 infants with post-vaccination titres, a higher LAZ score was associated with increased seropositivity (aRR 1.21 (95% CI 1.06, 1.38), P = 0.004), and higher birthweight was associated with increased IgA titres (0.45 (95%CI 0.18, 1.09) U/mL greater per 100 g gain in birthweight; P = 0.001).

**Conclusions:**

Infant ponderal and linear growth were positively associated with RVV immunogenicity, while concurrent administration of OPV was negatively associated with RVV immunogenicity. Together, these findings suggest that improving foetal growth and separating RVV and OPV administration are plausible approaches to increasing RVV immunogenicity.

## 1 Introduction

Oral rotavirus vaccines (RVV) have had a tremendous impact on the global burden of diarrhoeal disease since their introduction in 2006 [1]. However, rotavirus diarrhoea still underlies over 150,000 infant deaths each year and causes substantial morbidity, predominantly in low-income countries [2]. A major underlying reason is that currently available RVVs have consistently proven to be less efficacious in low-income settings. Across 11 Latin American countries and Finland, for example, two doses of monovalent RVV achieved efficacy against severe rotavirus gastroenteritis of 84.7% after a year of follow-up [3], [4], whereas for the same vaccine in South Africa and Malawi, one year efficacy fell to 61.2% [5]. This gap in vaccine performance presents a major obstacle to realising the full potential of RVV in regions where it could have the greatest impact.

Our understanding of why many children in low-income settings fail to achieve adequate protection from RVVs remains incomplete. Several explanations have been proposed, including greater strain diversity, maternal transplacental and breast milk antibody interference, vaccine formulation and malnutrition [6]. Malnutrition, which is common in the world’s poorest countries, manifests in several forms including impaired growth (length) and deficiencies in specific micronutrients. These are accompanied by a range of immunological deficits [7] which may impair immune responses to oral vaccine antigens. Intestinal factors in early infancy may also play a role in RVV underperformance including enteric infections [8], environmental enteric dysfunction [9], and microbiota dysbiosis [10]; however, studies exploring their contribution to RVV failure are heterogeneous [11]. Additionally, a number of trials have explored interventions to improve RVV performance, including adjuvants and changes in dose schedule, but with limited success [12].

Mathematical modelling has shown that reduced RVV immunogenicity is one of the principal factors compromising vaccine efficacy in LICs [13]. Indeed, RVV seroconversion has been shown to mirror trends in efficacy, with reduced rates in countries with high under-five mortality [14]. Moreover, recently published studies from LICs have described alarmingly low rates of RVV seroconversion, between 20 and 30% [15], [16], [17]. Progress in developing future interventions is contingent on a better understanding of the causes of poor immune responses to RVV in these settings. Previous research has characterised risk factors for poor immune response to parenteral vaccines [18], [19]; however, few studies have focused on oral vaccines or low-income settings. This study aimed to identify risk factors that independently predict seroconversion to RVV, to shed light on potential mechanisms reducing RVV efficacy in low-income countries.

## 2 Methods

### 2.1 Study design and participants

The SHINE trial design, procedures and outcomes have been reported in detail elsewhere (NCT01824940) [20], [21]. Briefly, SHINE was a 2x2 factorial, cluster-randomized trial across two districts in rural Zimbabwe, which tested the independent and combined effects of improved water, sanitation and hygiene (WASH) and improved infant and young child feeding (IYCF) on child length-for-age and haemoglobin at 18 months of age. Between November 2012 and March 2015, pregnant women were enrolled from clusters randomised to one of four arms: Standard-of-care (SOC), IYCF, WASH, or combined IYCF and WASH.

In May 2014, Rotarix^TM^, an oral monovalent rotavirus vaccine, was introduced to Zimbabwe’s Expanded Programme on Immunisation and given with oral polio vaccine at 6 and 10 weeks of age. All immunisation activities were undertaken at local clinics and not overseen by the trial; however, dates of vaccine receipt were recorded through maternal interview and transcription from child health cards. In addition, the SHINE trial protocol included a pre-specified objective to measure rotavirus immunogenicity among a subgroup of infants undergoing longitudinal specimen collection (https://osf.io/ad9zr/) [22].

Infants were eligible for the current analysis if they were HIV-unexposed, had received at least one dose of oral rotavirus vaccine and had available RVV immunogenicity data. We have previously shown that infants randomised to receive the WASH intervention had improved RVV immunogenicity [15], so infants in the WASH arms were excluded from this analysis to allow for unbiased evaluation of the predictors of RVV immunogenicity.

### 2.2 Outcomes of rotavirus vaccine response

Immunogenicity was determined using anti-rotavirus IgA titres, measured in cryopreserved plasma by enzyme-linked immunosorbent assay (ELISA) [23]. Whilst other measures of immune response to RVV exist [24], serum IgA seroconversion remains the correlate of protection with the greatest public health relevance [14], and was therefore selected as the primary outcome for the predictive model. Seroconversion was defined as a post-vaccine plasma.

concentration of anti-rotavirus IgA ≥ 20 U/mL in infants who were seronegative (<20 U/mL) pre-vaccination, consistent with the definition used in previous studies examining Rotarix^TM^ immunogenicity, including cohorts in the original efficacy trials [5], [25], [3], [26]. Secondary outcomes in the predictive model were seropositivity (defined as post-vaccine titre ≥ 20 U/mL, regardless of pre-vaccine titre) and anti-rotavirus IgA geometric mean titre (GMT).

### 2.3 Covariate data

We evaluated a broad range of demographic and programmatic factors in four categories: 1) variations in vaccine administration; 2) maternal and infant nutritional status; 3) history of diarrhoea prior to/around the first dose of RVV; and 4) household factors (Appendix, Table S1). Each category included variables that were selected based on biological plausibility and available data from the trial; those with little or no heterogeneity within the study population were excluded. Variables included were measured either at baseline (for mothers, this was at enrolment into the trial; for infants, this was at birth) or around the time of RVV receipt. Household wealth was assessed using a previously published composite score [27]. Infant anthropometry (weight, length and mid-upper-arm circumference) was measured at every postnatal visit; data from the 1 month postnatal visit were used in this anlaysis. Early initiation of breastfeeding was recorded at the 1-month visit by asking the mother how soon after delivery she first put her infant to the breast; responses ≤1 h were classified as early initiation. Exclusive breastfeeding (EBF) was assessed at the 1 month and 3 month visits using a tool previously developed in Zimbabwe [28], [29]. The rotavirus season in Zimbabwe was defined as 1st April–31st July based on national surveillance data from hospitals [30].

### 2.4 Statistical analysis

To model the relationship between potential predictors and rotavirus vaccine seroconversion, generalised estimating equations (GEE) with an exchangeable correlation structure were used, accounting for within-cluster correlation in the SHINE trial. A log binomial specification was used to estimate crude risk ratios for dichotomous outcomes. Candidate variables were selected in a 3-stage approach as described in previous risk factor analyses [31]. First (Step 1), independent associations with RVV immunogenicity were tested in univariable analyses; variables were retained based on a P value < 0.1. Second (Step 2), a multivariable regression model was fitted using all the variables selected in Step 1; those with a P value < 0.1 in the multivariable model were retained. Third (Step 3), a full model was developed to obtain adjusted RRs, which included all the variables retained in Step 2 as well as additional confounding variables selected *a priori* based on biological plausibility: concurrent oral polio vaccine (OPV) receipt with RVV dose 1; RVV dose 1 received during the rotavirus season; and breastfeeding status at time of RVV receipt. Separate models were built for each immunogenicity outcome (seroconversion, seropositivity and GMT). Anti-rotavirus GMT was left censored at 7.5 U/mL (assay limit of detection) using a Tobit regression model.

We undertook a sensitivity analysis accounting for variability in the timing of the pre- and post-vaccine blood draws, restricting the population to infants with titres measured at a narrower interval, defined as 0–14 days before the first dose of vaccine (for pre-vaccine titre) and 21–60 days after the last dose of vaccine (for post-vaccine titre).

All statistical analyses were performed using STATA version 14 (College Station, TX: StataCorp LP) and Prism v7 (GraphPad Software Inc., CA, USA).

## 3 Results

### 3.1 Baseline characteristics

**Fig. 1:**
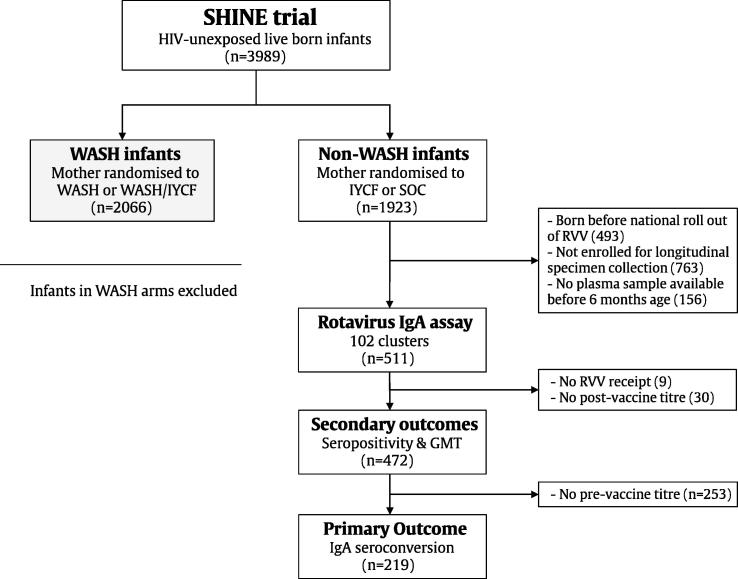
Consort flow diagram. The full trial flow, with additional detail, can be found in the Appendix, Fig. S1. WASH = Water, sanitation & hygiene; IYCF = infant & young child feeding; SOC = standard of care; RVV = rotavirus vaccine; GMT = geometric mean titre.

Among 5280 mothers enrolled in the SHINE trial, there were 3989 HIV-unexposed live births. Of these, 472 infants fulfilled the inclusion criteria for this analysis: all were in non-WASH arms with a complete record of RVV receipt and RVV immunogenicity data available. All 472 infants were included in analyses of seropositivity and GMT; 219 (46%) of these infants also had a pre-vaccine titre available and were included in the seroconversion outcome (Fig. 1). Infant characteristics are summarised in Table 1. Overall, 99% infants received both doses of RVV and the mean (SD) age at first dose receipt was 46.9 (10.8) days. Among 219 infants in the seroconversion analysis, 78 (36%) received their first RVV dose during the rotavirus season and 195 (96%) received their first dose concurrently with OPV. Around the time of vaccine receipt, 95% infants were still breastfeeding and 91% infants had been exclusively breastfed up until this time point. At one month of age, mean (SD) infant length-for-age Z-score (LAZ) was −0.6 (1.1) and mean weight-for-age Z-score (WAZ) was −0.2 (1.0).

**Table 1:** Characteristics of infants, mothers and households at baseline and around the time of RVV receipt.

	Primary outcome (seroconversion) (Infants N = 219)	Secondary outcome (seropositivity & IgA titre) (Infants N = 472)
*Infant baseline characteristics*		
Gender, % female	46.1	47.3
Birthweight, kilograms; mean (SD)	3.2 (0.4)	3.1 (0.5)
Low birthweight (<2.5 kg), %	5.2	7.4
Normal vaginal delivery, %	94.1	92.2
Early initiation breastfeeding (1st hour), %	90.1	91.0

*Maternal baseline characteristics*		
Age, years; mean (SD)	26.6 (6.3)	26.5 (6.5)
Parity, median (IQR)	2 (2, 2)	2 (2, 2)
Height, cm; mean (SD)	160.9 (5.6)	160.3 (6.1)
MUAC, cm; mean (SD)	26.9 (2.9)	26.6 (2.9)
Completed years schooling, median (IQR)	10 (9, 11)	10 (9, 11)
Maternal schistosomiasis*, %	26.6	26.3
Household size, median (IQR)	5 (3, 6)	5 (3, 6)
Improved floor in home, %	53.4	53.4
Electricity, %	3.3	4.2
Wealth Quintile:		
Lowest, %	15.2	16.6
Second,%	19.4	20.4
Middle, %	22.3	21.0
Fourth, %	24.2	22.1
Highest, %	19.0	19.9

*Infant characteristics peri-vaccination*		
Infant LAZ, mean (SD)	−0.6 (1.1)	−0.8 (1.2)
Infant WAZ, mean (SD)	−0.2 (1.0)	−0.4 (1.2)
Infant WHZ, mean (SD)	0.5 (1.5)	0.5 (1.5)
Concurrent OPV with RVV dose 1, %	96.1	94.7
EBF prior to RVV dose 1**, %	90.7	89.4
RVV dose 1 received in rotavirus season, %	35.6	38.6
Any diarrhoea prior to/around RVV dose 1, %	10.2	9.4
Both RVV doses received, %	99.1	99.4
Infant age at RVV dose 1 (days), mean (SD)	48.2 (11.4)	46.9 (10.8)

MUAC = mid-upper arm circumference, LAZ = length-for-age Z score, WAZ = weight-for-age Z score, WHZ = weight-for-height Z score, EBF = exclusive breastfeeding, RVV = rotavirus vaccine.

* Maternal schistosomiasis was defined as *S. haematobium* egg-positive and/or haematuria-positive.^#^ Diarrhoea based on maternal report and defined as 3 or more loose or watery stools in 24 h.

** Children were classified as exclusively breastfed if they consumed only breast milk and no other liquids or foods (except vitamins or prescribed medicines).

### 3.2 Risk factors for RVV seroconversion

Among 219 infants with seroconversion data, 43 (20%) successfully seroconverted and 176 (80%) failed to seroconvert to RVV (Table 2). Among infants who seroconverted, 53% were female, the mean (SD) birthweight was 3.2 (0.5) kg and 7% had low birthweight (<2.5 kg). Among seroconverters compared to non-seroconverters, there was no significant difference in the timing of pre-vaccine titre measurement (mean 11.0 versus 13.1 days prior to the first dose, respectively; P = 0.256) or post-vaccine titre measurement (mean 34.3 versus 29.3 days after the last dose, respectively; P = 0.151). Seroconverters were on average 3.6 days younger at the time of RVV receipt than non-seroconverters (mean (SD) 48.9 (12.2) days versus 45.3 (6.1) days, respectively; P = 0.065).

**Table 2:** Associations between infant, maternal and household factors and RVV seroconversion.

PRIMARY OUTCOME (seroconversion)	Non-seroconverter (Infants N = 176)^1^	Seroconverter (Infants N = 43)^1^	Univariable P value^2^	Multivariable Crude RR (95% CI); P value^3^	Fully adjusted model Adjusted RR (95% CI); P value^3^
*Characteristics at infant baseline*					
Gender, % female	44.3	53.5	0.295		
Birthweight, kilograms; mean (SD)	3.2 (0.4)	3.2 (0.5)	0.464		
Low birthweight (<2.5 kg), %	4.8	7.0	0.471		
Normal vaginal delivery, %	93.2	97.7	0.409		
Early initiation breastfeeding (1st hour), %	88.9	95.2	0.106		

*Characteristics at maternal baseline*					
Age, years; mean (SD)	26.8 (6.4)	25.8 (5.6)	0.429		
Parity, median (IQR)	2 (2, 2)	2 (2, 2)	0.684		
Height, cm; mean (SD)	160.7 (5.7)	161.6 (5.3)	0.444		
MUAC, cm; mean (SD)	26.9 (2.9)	27.1 (2.9)	0.488		
Completed years schooling, median (IQR)	9 (10, 11)	9 (10, 11)	0.359		
Maternal schistosomiasis, %	22.7 [163]	42.5 [39]	0.017	1.57 (0.87, 2.81); 0.131	
Household size, median (IQR)	5 (3, 6) [165]	5 (3, 6) [42]	0.275		
Improved floor in home, %	56.4	41.9	0.108		
SHINE wealth index, mean (SD)	0.11 (1.8)	0.19 (1.8)	0.844		

*Characteristics peri-vaccine*					
Infant LAZ, mean (SD)	−0.7 (1.1)	−0.3 (1.1)	**0.058**	1.26 (1.01, 1.57); 0.037	1.27 (1.04, 1.55); **0.021**
Infant WAZ, mean (SD)	−0.3 (0.9) [170]	−0.04 (1.0) [37]	0.161		
Infant WHZ, mean (SD)	0.5 (1.5) [169]	0.4 (1.4) [37]	0.594		
Concurrent OPV with RVV dose 1, %	97.6 [164]	89.7 [38]	**0.024**	0.34 (0.19, 0.59); **<0.001**	0.36 (0.19, 0.71); **0.003**
EBF prior to RVV dose 1, %	91.3	88.4	0.651		
Breastfeeding at time of RVV dose 1, %	94.2	92.9	0.997		
RVV dose 1 received in rotavirus season, %	34.7	39.5	0.628		
Any diarrhoea pre RVV dose 1, %	10.9	7.1	0.260		
Infant age at RVV dose 1 (days), mean (SD)	48.9 (12.2)	45.3 (6.1)	**0.064**	0.98 (0.95, 1.01); 0.119	

1 [N] provided in table for variables where missing data >5% (based on total number (219) with seroconversion status).

2 P values marked in bold if P < 0.1.

3 95% CI and P value marked in bold if P < 0.05.

In univariable analyses, four factors showed some evidence of an association with RVV seroconversion (P < 0.1) and were therefore retained in the model: maternal schistosomiasis in pregnancy; infant LAZ around the time of vaccination; infant age at time of RVV receipt; and concomitant administration of OPV (Table 2). In the multivariable model, only infant LAZ and concomitant OPV remained significantly associated with RVV seroconversion. In the final model, successful seroconversion was significantly higher among infants with a higher LAZ score (adjusted RR 1.27 (95% CI 1.04, 1.55), P = 0.021), and significantly lower among infants receiving OPV and RVV concurrently (aRR 0.36 (0.19, 0.71), P = 0.003). Household demographics including wealth, household size and maternal education were not predictive of RVV seroconversion. Seroconverters and non-seroconverters did not significantly differ in terms of breastfeeding status either before or around the time of RVV receipt.

### 3.3 Risk factors for RVV seropositivity and geometric mean IgA titre

Among 472 infants with secondary outcome data available, four factors were retained in the multivariable model to predict RVV seropositivity: higher birthweight; higher infant LAZ and WAZ around the time of vaccination; and younger infant age at RVV vaccination (Table 3). In the final model, the risk of seropositivity was significantly greater among infants with a higher LAZ score around the time of vaccination (aRR 1.21 (95% CI 1.06, 1.38), P = 0.004). In the analysis of geometric mean IgA titres, infant birthweight, LAZ and WAZ around the time of vaccination, and infant age at the time of RVV receipt had some evidence of an association with GMT in univariable analyses. Birthweight was the only factor retained in the final model. IgA titres were 0.45 (95%CI 0.18, 1.09) U/mL greater per 100 g gain in birthweight (P = 0.001; Table 4).

**Table 3:** Associations between infant, maternal and household factors and RVV seropositivity.

SECONDARY OUTCOME(seropositivity)	Seronegative (Infants N = 365)	Seropositive (Infants N = 107)	Univariable P value^1^	Multivariable Crude RR (95% CI); P value^2^	Fully adjusted model Adjusted RR (95% CI); P value^2^
*Characteristics at infant baseline*					
Gender, % female	46.3	50.5	0.416		
Birthweight, kilograms; mean (SD)	3.1 (0.5)	3.2 (0.5)	**0.009**	1.34 (0.85, 2.13), 0.207	
Low birthweight (<2.5 kg), %	7.9	5.7	0.459		
Normal vaginal delivery, %	91.5	94.4	0.336		
Early initiation breastfeeding (1st hour), %	90.5	93.2	0.427		

*Characteristics at maternal baseline*					
Age, years; mean (SD)	26.6 (6.6)	26.0 (6.3)	0.559		
Parity, median (IQR)	2 (2, 2)	2 (2, 2)	0.600		
Height, cm; mean (SD)	160.1 (5.9)	161.0 (6.7)	0.164		
MUAC, cm; mean (SD)	26.7 (2.8)	26.8 (3.1)	0.606		
Completed years schooling, median (IQR)	10 (9, 11)	10 (9, 11)	0.292		
Maternal schistosomiasis, %	24.3	33.0	0.151		
Household size, median (IQR)	5 (3, 6)	4 (3, 6)	0.606		
Improved floor in home, %	54.7	49.0	0.357		
SHINE wealth index, mean (SD)	0.1 (1.8)	0.1 (1.7)	0.965		

*Characteristics peri-vaccine*					
Infant LAZ, mean (SD)	−0.9 (1.2)	−0.5 (1.1)	**0.020**	**1.13 (0.98, 1.31), 0.098**	**1.21 (1.06, 1.38), 0.004**
Infant WAZ, mean (SD)	−0.4 (1.2)	−0.1 (1.1)	**0.043**	1.06 (0.88, 1.27), 0.567	
Infant WHZ, mean (SD)	0.5 (1.6)	0.5 (1.3)	0.726		
Concurrent OPV with RVV dose 1, %	95.6	91.7	0.148		
EBF prior to RVV dose 1, %	89.5	89.1	0.957		
Breastfed at time of RVV dose 1 receipt, %	91.3	90.3	0.733		
RVV dose 1 received in rotavirus season, %	37.8	41.1	0.587		
Any diarrhoea pre RVV dose 1, %	10.5	5.7	0.140		
Infant age at RVV dose 1 (days), mean (SD)	47.5 (11.3)	45.0 (8.8)	**0.027**	**0.98 (0.96, 1.00), 0.052**	0.98 (0.96, 1.00), 0.074

1 P values marked in bold if P < 0.1.

2 95% CI and P value marked in bold if P < 0.05.

**Table 4:** Associations between infant, maternal and household factors and geometric mean titre.

SECONDARY OUTCOME (GMT Units/mL)	Univariable P value^1^	Multivariable Beta (95% CI); P value^2^	Fully adjusted model Adjusted Beta (95% CI); P value^2^
*Characteristics at infant baseline*			
Gender, % female	0.457		
Birthweight, kilograms; mean (SD)	**0.001**	**3.50 (1.33, 9.19), 0.011**	**4.48 (1.84, 10.90), 0.001**
Low birthweight (<2.5 kg), %	0.158		
Normal vaginal delivery, %	0.482		
Early initiation breastfeeding (1st hour), %	0.135		

*Characteristics at maternal baseline*			
Age, years; mean (SD)	0.515		
Parity, median (IQR)	0.960		
Height, cm; mean (SD)	0.327		
MUAC, cm; mean (SD)	0.405		
Completed years schooling, median (IQR)	0.326		
Maternal schistosomiasis, %	0.196		
Household size, median (IQR)	0.971		
Improved floor in home, %	0.252		
SHINE wealth index, mean (SD)	0.975		

*Characteristics peri-vaccine*			
Infant LAZ, mean (SD)	**0.005**	1.22 (0.83, 1.79), 0.317	
Infant WAZ, mean (SD)	**0.025**	0.99 (0.65, 1.48), 0.944	
Infant WHZ, mean (SD)	0.811		
Concurrent OPV with RVV dose 1, %	0.192		
EBF prior to RVV dose 1, %	0.615		
Breastfed at time of RVV dose 1 receipt, %	0.784		
RVV dose 1 received in rotavirus season, %	0.905		
Any diarrhoea pre RVV dose 1, %	0.108		
Infant age at RVV dose 1 (days), mean (SD)	**0.013**	**0.96 (0.92, 1.00), 0.038**	0.96 (0.91, 1.01), 0.088

1 P values marked in bold if P < 0.1.

2 95% CI and P value marked in bold if P < 0.05.

### 3.4 Sensitivity analysis

Among 94 infants who had seroconversion status available with titres that were measured within a narrower window, only one factor (receipt of RVV concurrently with OPV) remained statistically significant in the final multivariable model (Table S2). Receipt of RVV with OPV was associated with a 69% reduced risk of RVV seroconversion (aRR 0.31 (95% CI 0.14, 0.70), P = 0.005).

## 4 Discussion

Overall, the immunogenicity of RVV was extremely low in rural Zimbabwe, with only one-in-five infants seroconverting following vaccination. Several factors emerged as independent predictors of RVV immune responses, including birthweight, length-for-age around the time of RVV receipt, and co-administration of OPV. Attained growth prior to vaccination was an important positive predictor across all measures of RVV immunogenicity: increased birthweight was associated with higher IgA titres, and increased infant LAZ at the time of rotavirus vaccination (approximately 6 weeks of age) was associated with higher rates of RVV seroconversion and seropositivity. Concomitant administration of OPV and RVV was negatively associated with seroconversion, in keeping with several previous studies from diverse settings [32], [33], [34], [35]. Collectively, these findings suggest that improving intrauterine growth and separating administration of RVV and OPV might be plausible approaches to improving oral vaccine immunogenicity.

Few studies have examined predictors of oral vaccine immunogenicity in low-income countries. A recent analysis explored risk factors for failed OPV seroconversion among children in MAL-ED [36] – a multisite birth cohort study in infants from eight diverse low- and middle-income countries across three continents [37]. Similar to our study, MAL-ED investigators used observational data of national vaccine programmes in real-world conditions as opposed to data derived from controlled, clinical vaccine trials. Children receiving more than three doses of OPV vaccine had higher odds of seroconversion to OPV serotypes 1 and 3. Poor socioeconomic status and high enteropathogen scores were associated with increased odds of failed OPV response. However, RVV immunogenicity data were not available in this study. Another study used data from Rotateq™ randomised controlled trials in Africa and Asia to conduct a *post hoc* exploratory analysis of infant characteristics as predictors of rotavirus vaccine efficacy [38]. Variables considered included infant age at first dose, gender, breastfeeding and nutritional status. When the African sites (Ghana, Kenya and Mali) were combined, infants receiving the first dose of RVV before 8 weeks of age had lower 2-year efficacy (23.7%, 95% CI −8.2, 46.3) than those vaccinated after 8 weeks (59.1%, 95% CI 34.0, 74.6). However, in individual country analyses, the difference was only significant for Ghana after one year of follow-up and not for the severe rotavirus gastroenteritis outcome. By contrast, our analysis focused on RVV immunogenicity, not vaccine efficacy. Whilst seroconversion is considered the best available correlate of protection, factors that compromise efficacy may not be the same as factors affecting immunogenicity. In our study, younger age at first dose of RVV was not predictive of failed RVV seroconversion. In fact, among the 86% of infants who received their first dose of RVV before 8 weeks, seroconversion rates were higher compared to infants given RVV beyond 8 weeks (22% versus 8%, respectively). However, age at vaccination was not retained as a predictor of RVV immunogenicity in our final models. Moreover, seroconverters were on average only 4 days younger at the time of RVV receipt than non-seroconverters, providing very little variability in age to observe an effect.

Previous studies exploring the association between infant nutritional status and oral vaccine responses have had mixed findings. In Pakistan, OPV seropositivity following multiple OPV doses (average = 10) was lower in stunted compared to non-stunted infants [39]; and in Bangladesh, underweight children had lower OPV3 titres at 12 months of age following three OPV doses [40]. In the *post hoc* analysis of Rotateq™ trial data, there was also some evidence in Ghana that underweight (WAZ < –2) at enrollment was associated with reduced RVV efficacy against severe rotavirus gastroenteritis over 2 years of follow-up. Efficacy was 19.0% (95% CI −75.1, 62.5) in underweight infants versus 67.9% (95% CI 41.3, 82.4) in those with normal weight (P = 0.06) [38]. However, RVV efficacy did not differ according to stunting or wasting status in the Ghana cohort. Similarly, RVV efficacy did not differ in those with and without malnutrition (defined as weight-for-age ≤10th centile) in a secondary analysis of data from a trial conducted in Brazil, Mexico and Venezuela [41]. A recent study among Kenyan infants reported reduced Rotarix™ effectiveness across three measures of malnutrition: underweight, wasting and stunting [42]. Notably, the numbers of children in the malnourished categories were small and the confidence intervals were wide, which limited the precision of their comparisons. Similarly, in our sub-study population, only 4% of infants were underweight (WAZ < −2) and 9% stunted (LAZ < −2) prior to RVV receipt. However, both LAZ score close to the time of vaccination and birthweight predicted outcomes of RVV immunogenicity. In SHINE, birth length was not measured, so the LAZ measured closest to vaccination was the first linear growth measurement among enrolled infants. In the whole SHINE cohort, 16% of infants were stunted at the time of this first length measurement [43], which is reflective of *in utero* stunting. The collective findings in the current analysis that both birthweight and pre-vaccine LAZ were associated with RVV immunogenicity suggest that poor intrauterine growth is an important determinant of RVV failure. Growth deficits already present at birth are known to be associated with a range of adverse health outcomes across the life-course [44], and may be associated with impairments in gut structure, function and mucosal immunity. For example, impaired mucosal integrity has been hypothesised to explain why postnatal HIV transmission via breastfeeding is increased among low birthweight Zimbabwean infants when compared to normal birthweight infants (adjusted hazard ratio 2.6 (95% CI, 1.4, 4.6)) [45]. Consistent with this hypothesis, mice with intrauterine growth restriction have abnormal intestinal architecture including fewer goblet and Paneth cells [46], which are integral to mucosal barrier function and innate immunity. Further mechanistic studies of oral vaccine immunogenicity in low birthweight infants are required. Overall, our data underline the importance of improving *in utero* growth and prioritising the antenatal period as a target for undernutrition interventions in vulnerable populations. A recent randomised trial in the Gambia showed that daily micronutrient and/or protein-energy supplements given to pregnant women (18.9% of whom were underweight) improved infant responses to the parenterally administered diphtheria pertussis tetanus (DPT) vaccine by 12 weeks of age [47]. Interventions designed to improve maternal nutrition may plausibly benefit oral vaccines as well, but trials are lacking.

Co-administration of OPV was a strong predictor of poor RVV seroconversion in this analysis. This is consistent with findings from a recent meta-analysis in which separating RVV and OPV administration was one of the few interventions that improved RVV seroconversion [12]. In our predictive model, seroconversion was 65% (95% CI 38, 80) less likely among infants receiving these vaccines together, compared to those who received them apart. Separate dosing was also the only factor that remained significantly associated with seroconversion in a sensitivity analysis, in which infant titres were measured during a narrower time interval before and after RVV receipt. These findings also support the results of an *in vitro* study showing that rotavirus replication in intestinal epithelial cells is reduced when mixed with other enteric viruses (astrovirus and enterovirus) [48]. However, the precise mechanisms for this viral interference remain poorly understood and warrant further study. Moreover, separate administration of OPV and RVV was not associated with either of the secondary immunogenicity outcomes for reasons that are uncertain. Overall, our data provide further evidence of a detrimental interference between these two live vaccines and justify consideration of programmatic strategies to separate RVV and OPV administration in EPI schedules. Indeed, this is already happening as a result of the Polio Endgame strategic plan, which includes the gradual phase out of OPV [49].

There are several strengths to this analysis. Although prior studies have examined RVV efficacy as an outcome, no studies to our knowledge have modelled predictors of RVV immunogenicity. This analysis considered a large baseline covariate dataset from mother-child pairs enrolled from two contiguous rural Zimbabwean districts, meaning the population was relatively homogeneous. In contrast, studies using multi-site trial data must account for major population differences between countries. The data also capture ‘real world’ conditions of vaccine administration, in contrast to vaccine efficacy trials. Nevertheless, there are also several limitations. Firstly, a large number of statistical tests were undertaken, increasing the risk of type 1 error due to false-positive associations. The small number of seroconverters also reduced the power to detect associations that may truly be present, introducing a risk of type 2 error. Secondly, there are several plausible predictors which were not considered in this analysis. Maternal IgG anti-rotavirus antibody, which can cross the placenta and plausibly interfere with infant immune responses to RVV, were not measured in SHNE but have previously been associated with reduced RVV seroconversion [50]. Population-level differences in histo-blood group antigen (HBGA) phenotypes may also influence immune responses to RVV. Although studies exploring infant HBGA status as predictors of RVV ‘take’ or conferred protection have had mixed findings [17], [51], [52], [53], maternal and infant secretor status would warrant further exploration in subsequent analyses. Finally, intestinal factors such as enteric infections and environmental enteric dysfunction were omitted from these predictive models although they have been examined in this population in separate analyses, and had no meaningful impact on RVV immunogenicity [54].

In summary, this study shows consistent associations between attained size at birth and infant responses to RVV, highlighting the potential importance of optimising fetal growth to improve oral vaccine immunogenicity. Our findings also recapitulate results from previous studies, describing interference between concurrent OPV and RVV. Although these findings do not fully explain the very low rates of RVV seroconversion in LICs, they offer further insights into the mechanisms behind oral vaccine failure and the potential targets for future intervention studies. Improving maternal nutritional status in pregnancy and infant growth prior to vaccination seem logical approaches to improve the immunogenicity of RVV based on our findings; and exploring programmatic strategies to separate RVV and OPV administration, without reducing vaccine uptake, warrants further evaluation.

## Funding

This work was supported by the Wellcome Trust [203905/Z/16/Z to JAC and 093768/Z/10/Z and 108065/Z/15/Z to AJP]. The SHINE trial was funded by the Bill and Melinda Gates Foundation [OPP1021542 and OPP113707]; UK Department for International Development (UK Aid); Swiss Agency for Development and Cooperation and US National Institutes of Health [2R01HD060338-06]. The study funders approved the trial design, but were not involved in data collection, analysis, interpretation, or manuscript preparation. The corresponding author had full access to the data and took the decision to submit for publication.

## Declaration of Competing Interest

The authors declare that they have no known competing financial interests or personal relationships that could have appeared to influence the work reported in this paper.
