# Supplementary material for: Predictors of oral rotavirus vaccine immunogenicity in rural Zimbabwean infants
Source: Vaccine. 2020 Mar 17;38(13):2870–8. doi: 10.1016/j.vaccine.2020.01.097 (PMC7065039; doi:10.1016/j.vaccine.2020.01.097)
Supplement: Supplementary data 1 [file mmc1.docx]

**Predictors of oral rotavirus vaccine immunogenicity**

**in rural Zimbabwean infants**

James A Church, Bernard Chasekwa, Sandra Rukobo, Margaret Govha, Benjamin Lee,

Marya P Carmolli, Robert Ntozini, Kuda Mutasa, Monica M McNeal, Florence D. Majo,

Naume V. Tavengwa, Beth D Kirkpatrick, Lawrence H Moulton,

Jean H Humphrey, Andrew J Prendergast

**Table of Contents for Appendix**

| **Supplementary tables** |  |
| --- | --- |
| 1. Table S1: List of variables to be considered in multivariable models | Page 2 |
| 1. Table S2: Sensitivity analysis (restricted window of pre- and post-vaccine titre) | Page 3 |
| **Supplementary figures** |  |
| 1. Figure S1: Detailed CONSORT flow diagram | Page 4 |

**Supplementary tables**

**Table S1:** List of variables to be considered in multivariable models according to four broad categories.

**Table S2:** Predictors of RVV seroconversion in a sensitivity analysis (where RVV titres measured at narrower interval).

P values marked in bold if P<0.1

95% CI and P value marked in bold if P<0.05

**Supplementary figures**

**Figure S1**: Detailed CONSORT flow diagram illustrating selection of infants in rotavirus IgA sub-study from overall population of infants enrolled in the SHINE trial.

^1^ 212 clusters were randomized, 53 in each of the four trial arms. After randomization, one cluster was excluded as it was determined to be in an urban area, one cluster was excluded as the VHW covering it mainly had clients outside the study area, and one more was merged into a neighbouring cluster based on subsequent data on VHW coverage. Three new cluster designations were created due to anomalies in the original mapping. For two of these, the trial arm was clear; the third contained areas that were in two trial arms, and was assigned to the underrepresented arm, resulting in 53 clusters per arm. All of this occurred before enrolment began. When enrolment was completed, however, there was one cluster (SOC) in which no women were enrolled, leaving a total of 211 clusters available for analysis.

^2^ Miscarriage, stillbirth or maternal death

^3^ No sample available, insufficient post-vaccine sample or assay failure

^4^ Insufficient or no pre-vaccine sample available

WASH = Water, sanitation & hygiene; IYCF = infant & young child feeding; SOC = standard of care; RVV = rotavirus vaccine; EED = environmental enteric dysfunction
